# Misleading measures in Vitamin D analysis: A novel LC-MS/MS assay to account for epimers and isobars

**DOI:** 10.1186/1475-2891-10-46

**Published:** 2011-05-14

**Authors:** Iltaf Shah, Ricky James, James Barker, Andrea Petroczi, Declan P Naughton

**Affiliations:** 1 School of Life Sciences, Kingston University, Kingston-upon-Thames, Surrey, UK; 2 School of Pharmacy and Chemistry, Kingston University, Kingston-upon-Thames, Surrrey, UK

## Abstract

**Background:**

Recently, the accuracies of many commercially available immunoassays for Vitamin D have been questioned. Liquid chromatography tandem mass spectrometry (LC- MS/MS) has been shown to facilitate accurate separation and quantification of the major circulating metabolite 25-hydroxyvitamin-D3 (25OHD3) and 25-hydroxyvitamin-D2 (25OHD2) collectively termed as 25OHD. However, among other interferents, this method may be compromised by overlapping peaks and identical masses of epimers and isobars, resulting in inaccuracies in circulating 25OHD measurements. The aim of this study was to develop a novel LC-MS/MS method that can accurately identify and quantitate 25OHD3 and 25OHD2 through chromatographic separation of 25OHD from its epimers and isobars.

**Methods:**

A positive ion electrospray ionisation (ESI) LC-MS/MS method was used in the Multiple Reaction Monitoring (MRM) mode for quantification. It involved i) liquid-liquid extraction, ii) tandem columns (a high resolution ZORBAX C18 coupled to an ULTRON chiral, with guard column and inlet filter), iii) Stanozolol-D3 as internal standard, and iv) identification via ESI and monitoring of three fragmentation transitions. To demonstrate the practical usefulness of our method, blood samples were collected from 5 healthy male Caucasian volunteers; age range 22 to 37 years and 25OHD2, 25OHD3 along with co-eluting epimers and analogues were quantified.

**Results:**

The new method allowed chromatographic separation and quantification of 25OHD2, 25OHD3, along with 25OHD3 epimer 3-epi-25OHD3 and isobars 1-α-hydroxyvitamin-D3 (1αOHD3), and 7-α-hydroxy-4-cholesten-3-one (7αC4). The new assay was capable of detecting 0.25 ng/mL of all analytes in serum.

**Conclusions:**

To our knowledge, this is the first specific, reliable, reproducible and robust LC-MS/MS method developed for the accurate detection of 25OHD (Vitamin D). The method is capable of detecting low levels of 25OHD3 and 25OHD2 together with chromatographic separation from the co-eluting epimers and isobars and circumvents other instrumental/analytical interferences. This analytical method does not require time-consuming derivatisation and complex extraction techniques and could prove very useful in clinical studies.

## Introduction

**Figure 1:**
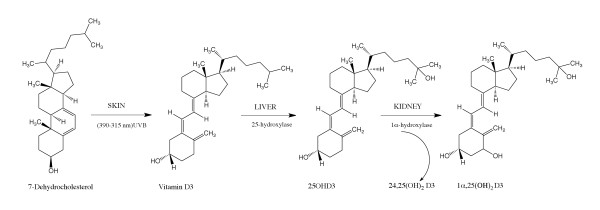
**Metabolic pathways for Vitamin D3 **[8-10].

Vitamin D plays a vital role in skeletal metabolism, calcium homeostasis, [1-3] and also in the functioning of the immune, cardiovascular, and reproductive systems [4,5]. Vitamin D deficiency leads to rickets and osteomalacia and is also associated with breast and colorectal cancers, multiple sclerosis, dementia, rheumatoid arthritis, diabetes, Parkinson's and Alzheimer's diseases [6,7]. Despite numerous reports, the associations of Vitamin D deficiency with health and diseases are subject to debate, partly owing to inadequacies in current approaches to measurement of serum levels. Depending on the source, Vitamin D is produced in two forms: Vitamin D2 and Vitamin D3, which differ by the presence of a double bond and methyl group on the aliphatic side chain. The issues involved in assessing Vitamin D status arise from the complexities of the metabolic pathways leading to a number of active forms. The complex metabolic pathway for Vitamin D3 is summarized in Figure 1.

Vitamin D3 is formed from its precursor 7-dehydrocholesterol in the skin by ultraviolet B light (medium wavelength, 290-315 nm) and Vitamin D2 originates from dietary sources together with some fraction of D3. In the liver, Vitamins D3 and D2 undergo hydroxylation reactions catalyzed by 25-hydroxylase, which leads to the formation of pharmacologically active metabolites 25OHD3 and 25OHD2 respectively (collectively termed as 25OHD). Further metabolism (in the presence of 1α,hydroxylase) in the kidney produces the pharmacologically active metabolites 1-alpha,25-dihydroxyvitamin-D3 (1α,25(OH)_2_D2) and 1-alpha,25-dihydroxyvitamin-D2 (1α,25(OH)_2_D3) along with the minor metabolite 24,25(OH)_2_D3 [8-10].

Since 25OHD has significant effects on health and wellbeing, there has been a substantial interest in improving the relevant analytical techniques [11-30]. Owing to a long serum half-life, measurement of total 25OHD (25OHD2 and 25OHD3) is the routinely used approach for assessing the total circulating Vitamin D status [10-14]. In immunoassay techniques, a measure of total metabolite concentration and equivalent detection of both 25OHD2 and 25OHD3 is challenging, as binding proteins show a higher affinity for 25OHD3 than 25OHD2 [15-18]. Reports have shown inter-laboratory and inter-method variations in results for Vitamin D determinations [19-21].

LC-MS/MS is currently the best technique available for the correct quantification of 25OHD3 and 25OHD2 [22,23] and it also has the capability to overcome most of the problems associated with protein binding assays. LC-MS/MS is a more favourable technique because sample derivatisation is not required, run time is very short and an internal standard is used which usually compensates for any matrix related and instrumental effects [24-32].

**Figure 2:**
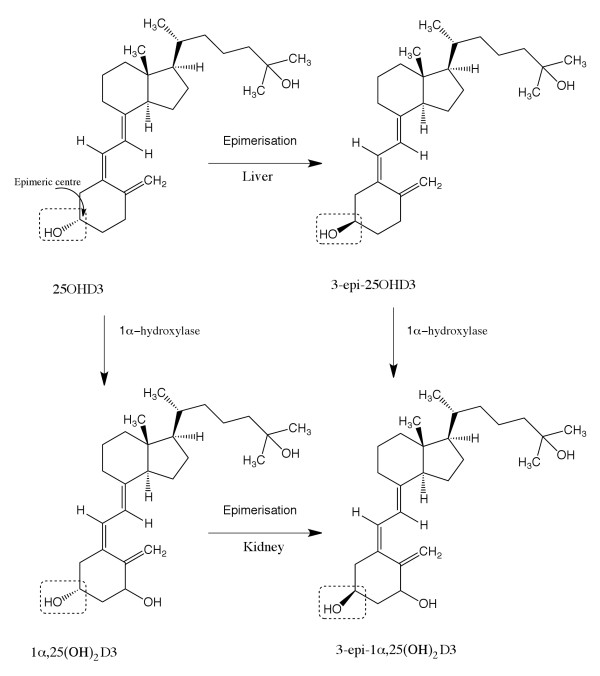
**Epimerisation and metabolic pathways for Vitamin D3 metabolites**. [adapted from reference [41]].

However, the LC-MS/MS approach is also subject to interferences [33-37]. Along with matrix related, instrumental and analytical interferences, endogenous 25OHD determinations have also been shown to suffer from epimeric and isobaric interferences [38-41]. Epimers are non-super imposable (or non mirror images) that only differ in the configuration at one carbon atom (Figure 2). Epimers and isobars are compounds with the same molecular weight as Vitamin D metabolites and form the same mass to charge parent and product ion pairs upon ionisation. Moreover, the separation of interfering epimers and isobars is also essential, because they can overlap chromatographically with Vitamin D metabolites or internal standard peaks and give false estimates of true Vitamin D levels. 25OHD3 is the most abundant Vitamin D metabolite in circulation and 3-epi-25OHD3 is the most prevalent epimer of 25OHD3. There are two compounds known to cause isobaric interferences in 25OHD analysis; 1α-hydroxyvitamin-D3 (1αOHD3), which is an exogenous pharmaceutical compound and 7α-hydroxy-4-cholestene-3-one (7αC4), which is an endogenous bile acid precursor [30,38-43].

The epimerization of 25OHD3 and 1α,25-(OH)_2_D3 results in the formation of 3-epi-25OHD3 and 3-epi-1α,25(OH)_2_D3 epimers respectively as shown in Figure 2. The epimers of 25OHD differ in configuration at third carbon atom (C-3) (shown by dashed highlights in Figure 2) that is attached to a hydroxyl group. Hydroxylation of 3-epi-25OHD3 forms 3-epi-1α,25(OH)_2_D3 [41].

The aim of this study was to develop a novel LC-MS/MS method that can accurately identify and quantitate 25OHD3 and 25OHD2 and chromatographically separate epimers and isobars.

## Materials and methods

### Blood samples

Blood samples (100 mL) were obtained from 5 healthy, male, Caucasian volunteers of age ranging from 22 to 37 years, weight ranging from 72.1-98.1 kg (mean weight 84.86 ± 11.3 kg) and height ranging from 165-190 cm (mean height 179.5 ± 8.6 cm). Blood samples were centrifuged at 1500 g and serum was extracted. The serum samples were frozen individually in labelled, amber, plastic vials [29]. To minimise assay variations, a multilevel serum calibrator set (Chromsystems, Germany) was used for preparation of calibration curves and quality controls. The lyophilised calibrators (based on human serum) consisted of 3 high and 1 low-level calibrators and were handled in the same manner as volunteer specimens. According to the assay procedures, the calibrators were analysed along with routine samples to meet the standards outlined by National Institute of Standards and Technology (NIST) [44,45].

### Standards and reagents

GV-65C (3 mL syringe) mixed bed cation exchange columns were obtained from Biochemical Diagnostics, (New York, USA), Bond Elut-SI, Bond Elut Plexa, Bond Elut LMS, Bond Elut PPL, SampliQ OPT and SampliQ DVB solid phase extraction cartridges were purchased from Agilent Technologies (Cheshire, UK). 25OHD3, 25OHD2, 1-α-hydroxyvitamin-D3 (1αOHD3), 3-epi-25OHD3, hexane, isopropanol, methanol, dichloromethane, deionised water, formic acid, acetonitrile, ammonium hydroxide, pentane and ether were obtained from Sigma Aldrich (Poole, UK). 7-α-hydroxy-4-cholesten-3-one (7αC4) and stanozolol-D3 (internal standard) were obtained from LGC standards (Teddington, UK). All chemicals and reagents were of HPLC grade.

### Preparation of standards and samples

Stock solutions of all analytes were prepared in methanol to obtain a concentration of 1 mg/mL and stored in amber vials at -20°C in the dark. Under these conditions, the stock solutions were found to be stable for 3 months. The solutions were kept in the dark to minimise light induced degradation of Vitamin D [46,47]. Working solutions were made in methanol by serial dilution of stock solutions. Working internal standard solution was also prepared by diluting the stock solution of internal standard with methanol to a final concentration of 1 μg/mL. Calibrators and internal standard solutions made in-house were stable for 2 weeks when stored at -20°C. The lyophilised serum calibrators were reconstituted in HPLC grade water and allowed to stand for 10 to 15 minutes at room temperature. The vials were then swirled to dissolve the contents until homogeneity. The lyophilized reference serum calibrants were stable for 3 months when stored at -20°C [46,47]. The calibration curves and quality controls were prepared from the multilevel reference calibrator set in the range 0.5 to 84.4 ng/mL concentrations, respectively. Different methods and conditions for sample pretreatment and extraction were undertaken to optimise recovery, specificity and signal to noise ratio. The sample pretreatment and extraction methods [12-32] were adopted as follows.

### Sample pretreatment

Serum samples were thawed; vortex mixed and equilibrated at room temperature for 15 minutes and 25 μL of working solution of stanozolol-D3 (internal standard) was added to all samples. Formic acid (2 M, 50 μL) was added and the resultant solution vortexed. Then 3 mL of methanol/isopropanol (1:1, v/v) mixture was added and vortexed to release the protein bound analyte and to promote protein precipitation during a 15 minutes incubation at 4°C. The suspended matter was removed after centrifugation at 3500 g for 5 minutes at 4°C. The supernatants were transferred to clean amber glass tubes. The remaining solution was subjected to further sample purification, as follows.

### Liquid-Liquid extraction (LLE)

Different solvent mixtures were tested for liquid-liquid extraction: namely; heptane, methanol, propanol, dichloromethane, acetonitrile and hexane. Hexane/dichloromethane (1:1, v/v) mixture was found to give optimum extraction recovery. For liquid-liquid extraction a 3 mL hexane/dichloromethane mixture (1:1, v/v) was added to the supernatant after protein precipitation and the solution was vigorously vortexed for 1 minute and then centrifuged at 3500 g for 5 minutes at 4°C. The supernatant layer was transferred to clean, amber, glass tubes. The residual lower layer of the serum sample was further extracted twice with the hexane/dichloromethane (1:1, v/v) mixture. The organic phase obtained was pooled and dried under a gentle stream of nitrogen at room temperature. It was then reconstituted in 200 μL of HPLC grade methanol/water (1:1, v/v) mixture. Solid phase extraction (SPE) was also tested as an alternative to the liquid-liquid extraction technique.

#### Solid phase extraction (SPE)

**Figure 3:**
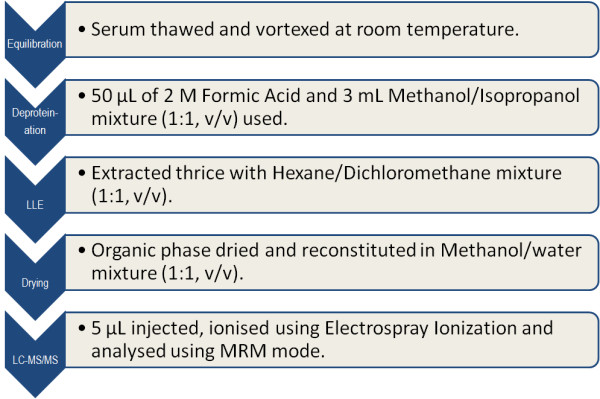
**Schematic diagram showing sample purification and analysis**.

A range of different sorbent packing materials was tested for SPE purification: namely, Biochemical Diagnostics's GV-65C cation exchange columns and Agilent Technologies range columns (Bond Elut Plexa, Bond Elut LMS, Bond Elut PPL, Bond Elut-SI, SampliQ OPT and SampliQ DVB). Agilent Bond Elut-SI silica gel (3 mL, 500 mg) was found to give the optimum elution recovery. For SPE extraction, the cartridges were first activated with hexane, followed by addition of methanol and equilibration with water. The samples were then loaded onto the SPE cartridges and extracted using gravity. The cartridges were washed with 3 mL water and then 3 mL methanol before drying by applying a negative pressure for 5 minutes in a vacuum manifold. Different eluents were tried for best elution recovery: namely; methanol, acetonitrile, 2% formic acid in methanol, methanol/propanol mixture (1:1, v/v), NH_4_OH (2 M, 50 μL/acetonitrile 3 mL) and ether/hexane mixture (1:1, v/v). The optimum elution recovery was achieved with 3 mL ether/hexane mixture (30:70, v/v). The residues were dried under a gentle stream of nitrogen at room temperature and reconstituted in 200 μL of methanol/water mixture (1:1, v/v). The recovery of the LLE method was found to be 11% greater than the corresponding SPE method. Thus liquid-liquid extraction technique was preferred over solid phase extraction. A schematic representation of serum sample purification for analysis using LC-MS/MS is shown in Figure 3.

### LC-MS/MS procedure

**Figure 4:**
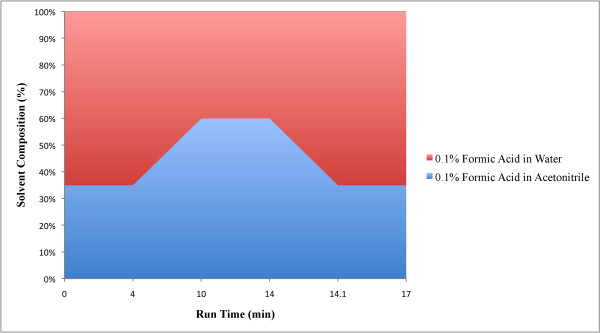
**Mobile phase gradient composition: solvent A (0.1% formic acid in acetonitrile) and solvent B (0.1% formic acid in water)**.

The LC-MS/MS system consisted of an autosampler (PAL-CTC Analytics, Switzerland), a turbomolecular pump (1100 series, Agilent Technologies, USA) and a Triple Quadrupole mass spectrometer (PE-SCIEX API-3000, Applied Biosystems Division of MDS Health Group Ltd, Canada). Analyst software version 1.4.2 (AB SCIEX) was used for results acquisition and quantitation. A 5 μL aliquot of the sample was injected into the LC-MS/MS system for analysis. Samples were analysed in the low light conditions, as it has been observed that Vitamin D has a greater stability under these conditions [46,47]. An Agilent microbore ZORBAX SB-C18 RRHD column (2.1 mm × 100 mm, 1.8 μm) was used in tandem prior to an ULTRON ES-OVM Chiral column (2 mm × 150 mm, 5 μm) for analysis. A low dispersion inlet filter (frit diameter 2.1 mm) was installed prior to the Agilent column to minimise external band spreading and improve peak shapes. A chiral guard column (ULTRON ES-OVM) of specification (4 mm × 10 mm) was also placed post filter and prior to the Agilent column to minimise interferences caused by instrumental and matrix contaminants. The column oven temperature was maintained at 40°C. Different mobile phases and their gradient compositions were tested for best results. Optimum peak resolution was achieved using 0.1% formic acid in acetonitrile (solvent A) and 0.1% formic acid in water (solvent B). Gradient composition of the two solvents is shown in Figure 4. The total flow rate through the columns was 200 μL/min.

The mass spectrometer was operated in positive electrospray ionisation (ESI) mode at a spray voltage of 5000 V and capillary temperature of 450°C. The generated protonated molecules of 25OHD3, 25OHD2, 3-epi-25OHD3, 1αOHD3, 7αC4 and stanozolol-D3 (internal standard) were used as precursor ions for collision activated dissociation (CAD) into product ions in MS-MS analysis.

## Results

Calibration curves and quality controls at low, medium and high concentration levels were prepared from the multi-level reference standards with known amounts of 25OHD. The assays were validated for specificity, recovery, linearity and intraday/interday precision and accuracy. The lower limit of detection (LLOD) for all analytes was found to be 0.25 ng/mL. The LLOD was determined by decreasing the analyte concentrations until a response equivalent to 3 times the background level was observed. The relative extraction recoveries at a final concentration of 50 ng/mL were determined by comparing the representative peak areas of extracted analytes (*N *= 6) to un-extracted analytes. The un-extracted analyte solutions were prepared in methanol. Validation results are summarised in Table 1.

**Table 1:** Summary of assay validation results.

Compounds	Linear range (ng/mL)	LLOD (ng/mL)	Recovery (%) at 50 ng/mL	Concentration (ng/mL)	**r**^**2**^	Intraday (%CV)	Interday (%CV)	Accuracy (%)
25OHD3	0.5-84.4	0.25	95	12.5	0.9999	2.1	12.1	97.5
				25		4.6	6.7	92.9
				50		7.1	4.1	92.2
25OHD2	0.5-84.4	0.25	88	12.5	0.9996	7.5	8.1	99.4
				25		9.1	8.6	97.5
				50		6.8	5.5	101.2

Under- or over-estimation of actual 25OHD3 concentrations may occur owing to co-eluting epimers (e.g.3-epi-25OHD3) and isobars (e.g.7αC4) [39-42]. The use of high resolution microbore ZORBAX column in tandem with chiral ULTRON column not only facilitated accurate determination of the analyte ions but also chromatographically separated all isobars and epimers from 25OHD co-eluting peaks.

Multiple reaction monitoring (MRM) mode was used for the analysis of 25OHD2 and 25OHD3. The MRM parameters were optimised using direct infusion of 0.1 μg/mL solutions of standard compounds. The MRM sequence consisted of two periods executed sequentially to monitor different transition pairs using parameters optimised for each period. The most abundant MRM ion transitions for each analyte were acquired using the conditions given in Table 2.

**Table 2:** Retention times, MRM transitions and ESI conditions of analytes.

Analytes	Retention time (min)	Transition (m/z) Precursor→ Product	Collision energy (eV)
		401.3 → 383.1	36
25OHD3	13.5	401.3 → 365.1	41
		401.3 → 159.2	46
		413.3 → 395.5	46
25OHD2	11.1	413.3 → 377.2	51
		413.3 → 355.5	53
		401.3 → 383.1	36
3-epi-25OHD3	13.1	401.3 → 365.1	41
		401.3 → 159.2	46
		401.3 → 383.1	36
1αOHD3	11.1	401.3 → 365.1	41
		401.3 → 159.2	46
		401.3 → 383.1	36
7αC4	10.5	401.3 → 365.1	41
		401.3 → 159.2	46
Stanozolol-D3 (I.S)	3.09	332.2 → 81.2	42

According to a joint position statement issued by British Association of Dermatologists, Diabetes UK, the Multiple Sclerosis Society UK, the National Heart Forum UK and the National Osteoporosis Society UK, the threshold level quoted for Vitamin D deficiency is ≤ 10 ng/mL (≤ 25 nmol/L) [48]. Individuals with ≤ 10 ng/mL of 25OHD are regarded as Vitamin D deficient. Some experts define Vitamin D deficiency as 25OHD levels ≤ 20 ng/mL [49-51]. However, the levels of Vitamin D can vary between individuals and currently there is no standard set for optimal Vitamin D levels [49-51]. Amongst the five samples analysed by LC-MS/MS, three were found to be below the effective 25OHD levels. Vitamin D serum results are shown in Table 3.

**Table 3:** Vitamin D serum results.

Volunteers	Age	25OHD3	25OHD2	Total 25OHD	Total 25OHD	25OHD levels*
		(ng/mL)	(ng/mL)	(ng/mL)	nmol/L	
1	21	2.8	7.0	9.8	24.0	Low
2	41	11.3	5.2	16.5	40.7	Normal
3	24	3.4	3.3	6.7	16.4	Low
4	33	15.2	2.5	17.7	43.8	Normal
5	38	6.5	2.5	9.0	22.1	Low

*According to ref. 48.

**Figure 5:**
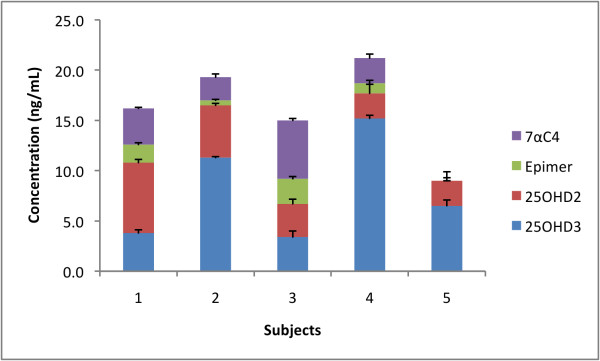
**Vitamin D levels and co-eluting epimers and isobars in five volunteers**.

The levels of potential interference owing to epimers and isobars are shown in Table 4. Clearly, if not chromatographically separated, these interferents could make a major contribution (in the range of 14 to 55%) to the total measured level of Vitamin D. The comparison between levels in Tables 3 and 4 reveals that where epimers and isobars are not accounted for, the measurements of Vitamin D levels are inflated to appear as normal. In these cases a diagnosis of deficiency may be missed as illustrated in Figure 5. The percentage of interfering epimer and isobars in serum, are detailed in Table 4. The isobar (1αOHD3) was not detected in any samples.

**Table 4:** The percentage of interfering epimer (E) and isobars (I) in serum.

Volunteers	25OHD	E		I		25OHD+E+I	Total E+I	% E+I
	ng/mL	ng/mL	%	ng/mL	%	ng/mL	ng/mL	of total
1	9.8	1.8	11.8	3.6	23.7	15.2	5.4	35.5
2	16.5	0.5	2.6	2.3	11.9	19.3	2.8	14.5
3	6.7	2.5	16.7	5.8	38.7	15.0	8.3	55.3
4	17.7	1	4.7	2.5	11.8	21.2	3.5	16.5
5	9	0	0.0	0	0.0	9.0	0	0.0

E = 3-epi-25OHD3, I = 7αC4 and1αOHD3

## Discussion

These results confirm the complexities involved in measuring Vitamin D status and reinforce the need for the uniform adoption of improved accurate assays. Measurements of 25OHD in human serum using competitive immunoassays are difficult owing to lipophilicity/tight binding to the Vitamin D-binding protein (DBP), even at very low serum concentrations [15]. In addition, immunoassays for 25OHD have been reported to cross-react with 24,25(OH)_2_D3, an intermediate product during the formation of 1,25(OH)_2_D3 in the kidney [16,17] (Figure 1). Many commercial immunoassays can only measure 25OHD3 and are not suitable to monitor supplementation with Vitamin D2, which is derived from plant sources and widely used in many countries for fortification of foods [18]. Hence, LC-MS/MS analysis is preferred but it has been shown that it is also subjected to interference issues [19-28]. In our new method, the use of an electrospray ionisation technique has overcome the problems associated with in-source transformation of 25OHD3-sulfate and other metabolites to 25OHD3 which may occur when using atmospheric pressure chemical ionisation mode [29].

The use of tandem column technology, an optimised mobile phase composition and a modified extraction method not only separate the epimers and isobars but also eliminates the interferences caused by early eluting salts (e.g. sodium) and the late eluting phospholipids, which may interfere with analyte ionisation [29-34]. Moreover, the installation of a low dispersion inlet filter together with a chiral guard column also minimises the interferences resulting from early eluting amino acids and late eluting xenobiotics. The column also separates interfering compounds present at higher concentration which have been shown to overlap or share exact mass transitions with 25OHD e.g. detergents and phthalates etc [29-31].

The separation of epimers and isobars from the target analyte is critical because they can overlap 25OHD peaks and form the same masses upon ionisation, thus compromising the true status of 25OHD in circulation [38-43]. The epimer of 25OHD3 is known to have the same effects on suppressing parathyroid hormone (PTH) secretion, but it has negligible calcium-producing effects [40,41]. Recently, it was concluded that the absence of external standardisation for the 25OHD assay might lead to greater variations and false results [33]. To minimise the inter-laboratory and inter-method variations in our LC-MS/MS analysis, we have used reference materials for preparation of our calibrants and quality controls, along with the introduction of a new internal standard. The latter was required as, when using the internal standard hexadeuterated-25OHD3, certain fragment transitions of the parent ions could lead to greater isobaric interferences such as the transition 407.7 > 389.7 [37]. Stanozolol-D3 as a new internal standard prevents isobaric interferences by using the transition 332.2 > 81.2.

To date, only one transition of the parent ion to product ion has been investigated, instead of our three-precursor ions to fragment ions transitions (according to the qualifier-qualifier principle) [29]. To our knowledge, this is first time that three transitions of precursor ions to product ions have been used for quantification of 25OHD3, 25OHD2, 3-epi-25OHD3, 1αOHD3 and 7αC4 as shown in Table 2. The sum of the three transitions not only increases the sensitivity of the assay but also minimises isobaric interferences.

To summarise, this is the first LC-MS/MS method for the determination of 25OHD which includes separation and quantification of epimers and isobars. It is a fast liquid-liquid extraction method, which does not require complicated derivatisation procedures, hence reducing assay time and variability. This method can quantify the different 25OHD forms accurately, which is difficult to achieve using immunoassay methods. This methodology can be used for accurate blood testing to prevent falsely elevated Vitamin D levels being reported.

## Conclusions

The LC-MS/MS method was free from all types of interferences arising due to epimeric, isobaric, instrumental and matrix components, which may interfere with analyte ionisation. Thus the LC-MS/MS method provides a robust, specific, reliable and reproducible technique as a solution to the problems identified in relation to current assays for Vitamin D. Removal of uncertainties in Vitamin D measurement, is required in order to progress the current understanding of the roles of Vitamin D in health and disease via more rigorous clinical trials [7].

## Competing interests

The authors declare that they have no competing interests.

## Authors' contributions

AP and DPN initiated the study. The method development was conducted by IS with contributions from DPN and JB. RJ provided samples for analysis. All authors contributed to the study design, preparation of the manuscript and have read and approved the final version.
